# Supplementary material for: Measurement of inferior colliculus volume based on MRI image stacks and its relationship with age and hearing status
Source: PLoS One. 2025 Jan 28;20(1):e0317363. doi: 10.1371/journal.pone.0317363 (PMC11774353; doi:10.1371/journal.pone.0317363)
Supplement: Attachment — Submitted filename: response to reviewers.docx [file pone.0317363.s002.docx]

PONE-D-24-15625	Response to Reviewers

Measurement of inferior colliculus volume based on MRI image stacks and its relationship with age and hearing status

Dear Prof. Sakurai and dear reviewers,

thank you for your constructive recommendations which are most helpful in improving this manuscript.

Academic Editor:

1) We have adapted **the format of our manuscript** to the PLOS ONE´s style requirements.

We have stated the references in Vancouver style. Also, we have added supporting information (all raw data under "S1 Table"); the caption for this has been added at the end of the manuscript.

2) **In the online submission form you indicate that your data is not available for proprietary reasons and have provided a contact point for accessing this data.**

The data has now been made available in the supplementary information "S1 Table".

3) **Please amend either the title on the online submission form (via Edi Submission) or the title in the manuscript so that they are identical.**

This has been done.

4. **Please include a caption for figure 5.**

The caption for figure 5 is in the lines 252-255 of the manuscript.

Reviewer # 1

**You mentioned that all patients have PTA or BERA. However, it is crucial to specify the type of BERA that was employed, as the BERA-click does not provide data that is comparable to PTA. Therefore, examine this item and substitute the procedure name for BERA-specific frequency.**

For the present exploratory retrospective analysis of the relation of hearing status and IC volume we noticed early during the study the heterogeneity of the subjects with regard to age and hearing status (e.g. Fig. 4). Thus, we focused on a coarse classification of hearing loss that can be achieved by evaluating standard clinical PTAs and BERAS performed in the audiology section of the Regensburg ENT clinic.

To obtain frequency specific information we recorded in Regensburg the response to short tone bursts which were used to evaluate the hearing status. For measuring the interpeak-latences I-V click stimuli were used.

We have added a sentence concerning this issue in lines 137-139 of the manuscript.

**PTA – indicating the values and equipment that are used.**

See Raw data (S1 Table) that are now provided in “supplementary data” and give the thresholds obtained at the PTA test-frequencies and for the BERA stimuli.

The equipment used for PTA: Madsen Astera, Co. Otometrics/Münster-Germany

The equipment used for BERA: Navigator Pro, Bio-Logic/Natus, Model 580-NVBOX1156A, Worthing, UK.

**The BERA frequency specifies the values and equipment that are employed. Before the specified frequency was reached, BERA was executed–click?**

To obtain frequency specific information we recorded in Regensburg the response to short tone bursts which were used to evaluate the hearing status. For measuring the interpeak-latences I-V click stimuli were used.

**Why was 5kHz employed? This frequency is not of great significance within an audio battery.**

For the BERA values in this study, the Navigator Pro-machine was used which had been preset for the examination with click-stimuli using the upper frequency limit at 5kHz.

In our newer BERA equipment (Eclipse by Interacoustics, Middelfart, Denmark) the upper frequency limit for clicks is now 4kHz.

However, as this is a retrospective study, the examination frequency cannot be changed to 4kHz. Consequently, high frequency loss in the BERA measurements was determined in the majority of earlier data by 5kHz and in more recent measurements with a 4kHz setting.

**It is strongly recommended that auditory loss be classified in accordance with the guidelines of the World Health Organization (2019). And that the categories be divided by this classification.**

Our classification is in close accordance with the classification mentioned above. It combines the grades 0 (no impairment, <25dB) and 1 (slight impairment, 26-40dB) as category 1 (normal to mild hearing loss) and grade 4 (profound impairment including deafness, >81dB) as category 3 (severe hearing loss or deafness) as listed in lines 148-154.

**I would have appreciated the opportunity to review a prior study that examined the number of participants who would be considered reliable for this study.**

Since this exploratory retrospective study was, to our knowledge, the first systematic trying to correlate the relation of IC-volume with other factors like age or hearing status, these data can provide the basis to estimate the required sample size for more detailed future studies.

**Note that the size of the letters is greater than the remainder of the manuscript on page 10, lines 182 to 184.**

This formatting mistake has been corrected. Due to corrections in the manuscript the lines have shifted to 189-191.

**It is essential to mention in a specific section of the manuscript that the PTA is the standard for diagnosing auditory loss in children who are capable of responding appropriately to the elderly.**

PTA was used in older children and adults when possible. BERA was used in babies when a PTA could not be reliably measured. In some deaf children over 1 year a BERA was performed in the course of Cochlear Implant diagnostics.

This we now mentioned in the methods part lines 132-137:

The PTAs are the standard for diagnosing auditory loss in children who are capable of responding appropriately to the examiner. PTA was used in 67 children and adults and determined air-conduction thresholds using 9 frequencies (125Hz to 8kHz). The BERA was used in 56 young children (aged 3 months to 4 years) in whom a PTA could not be reliably measured, and at least 3 frequencies were included (500-1000Hz, 2kHz, 5kHz). In some deaf children over 1 year of age, a BERA had been performed in the course of cochlear implant diagnostics.

**In the discussion, the first sentence indicates that hearing loss is more prevalent after the age of 70. I suggest reading additional articles, as there is already evidence that it occurs more frequently today.**

The National Institute on Deafness and Other Communication Disorders states in their Quick Statistics about Hearing, Balance & Dizziness Report which was updated Sept. 20, 2024, that among adults ages 20-69 who report 5 or more years of exposure to very loud noise at work, about 18% have speech-frequency hearing loss in both ears. About 5% of adults ages 45-54 have disabling hearing loss. The rate increases to 10% for adults ages 55-64. 22% of those ages 65-74 and 55% of those who are 75 and older have disabling hearing loss.

These numbers do not differ so much from the ones stated in our discussion.

Another study from 2010 looked at hearing thresholds from 1999-2004 and compared them with thresholds from 1959-1962 ( Hoffman HJ et al. Americans hear as well or better today compared with 40 years ago: hearing threshold levels in the unscreened adult population of the United States, 1959-1962 and 1999-2004. Ear Hear 2010 Dec;31(6):725-34.) They found that prevalence of overall hearing impairment was significantly lower in 1999-2004 than in 1959-1962.

However, the population worldwide is becoming older and, therefore, age-related hearing loss can be found more frequently than in the past.

**Additionally, I suggest that the discussion regarding prevalence and incidence be more in-depth, including, for instance, the fact that adolescents and children tend to lose their hearing even more rapidly as a result of the use of hearing aids. The initial paragraph is extremely rudimentary and inadequate.**

The aim of this study was to examine the volumes of the inferior colliculus and relate them with age and hearing loss. A discussion regarding prevalence of hearing loss and the impact of the use of hearing aids on hearing in adolescents would be suitable for a review study but not for this retrospective analysis, which cannot find answers for the changes in prevalence of hearing loss.

**In terms of the IC volume, it was determined that two individuals exhibited values that were higher. I suggest that they draw conclusions about this. What was the profession of these individuals? Were they musicians? In conclusion, they must better package this item.**

This is an interesting point and we looked up these two cases.

Both patients with the high IC volumes happened to be deaf children (3 months and 4 years old), so they were certainly not musicians. We could not draw any sound conclusions from the results.

**Furthermore, when discussing the IC volume in relation to left/right, they also mentioned left/right-handedness very appropriately. However, did you analyze this variable?**

Unfortunately, we have no records of the handedness.

**It would be an extremely significant point in this study. Given the impossibility of this analysis, as it is a retrospective study, I suggest that it be included as a constraint on the study.**

The following sentence was added to the discussion lines 387-388

It is a constraint of this study, that handedness cannot not be considered in the discussion of the IC volume in relation to right/left sides (lateralization of hearing), as there were no records available.

**I suggest that the study's limiting factors, which encompass a multitude of other factors, be updated, and that new studies be proposed.**

This sentence was added at the end of the discussion line 416-418:

Since this exploratory retrospective study was, to our knowledge, the first systematic trying to correlate the relation of IC-volume with other factors like age or hearing status, these data can provide the basis for focused study designs comparing age matched groups with well-defined hearing status and for the estimation of the required sample size for more detailed future studies.

**Furthermore, it is not possible to generalize and assert that there is no symmetry, which would imply that this was observed in the current study with a reduced number of participants.**

A Wilcoxon paired sample test comparing left and right IC volume of the 123 subjects found no systematic significant difference or systematic asymmetry between both sides (p = 0.865).

We do not state in the manuscript that “there is no symmetry”, rather we describe that the data “show no systematic difference … between both sides.” and this is also reflected in the discussion lines 381 – 386.

**The same applies to the other apprehensions. This study must be replicated with an appropriate number of participants, as well as more clearly defined evaluation procedures.**

We agree that our retrospective explorative analysis cannot unequivocally answer many detailed questions. But it provides the basis for the focused design of future studies with adequate estimates of required sample size.

Reviewer #2

**I thank the editors of PLOS One for the opportunity to review this article titled “Measurement of inferior colliculus volume based on MRI image stacks and its relationship with age and hearing status”. In this article, the authors report a study into the association between inferior colliculus volume (measured retrospectively using MRI) and patient age, sex, and hearing status. The article reviews the prevalence and impact of age-related hearing loss, the role of the inferior colliculus, and the phenomenon of lateralisation in the brain. The authors address the knowledge gap of whether there is any lateralisation of the IC in humans, and how IC volume correlates with age and hearing loss. The article concludes that IC volume is age dependent and symmetrical, and speculates that IC volume may be associated with age.**

**My major concerns in relation to the article are regarding the choice of participant groups and the statistical comparisons made. The group comparisons performed do not seem to be the most intuitive and as such the reader is left wondering whether further tests were performed but not included in the article as they were non-significant. More justification needs to be given and more direct tests need to be performed and reported.**

This is an explorative analysis of retrospective data to evaluate the feasibility if IC-volume measurements in routine clinical MRIs. In addition, we attempted to use the resulting volume data to test a number of hypotheses that appeared fairly relevant to us in the context of auditory research:

- Effect of age
- Left – right symmetry
- Effect of hearing loss

We have not tested additional hypotheses (e.g. effect of left-right handedness on IC symmetry, or effect of “musicality” on IC volume, or the effect of smoking) because we did not have these data. We did not selectively report “significant” results and “drop insignificant” results, we explored the hypotheses listed above and presented the results based on the available data base.

**I worry a little about the slight over-statement in the abstract “suggesting that severe hearing loss may be associated with a reduced volume of the inferior colliculus in aged humans”. This seems much diluted in the article, whereby the and of the introduction states that “a definite answer for question 4 was not achieved”**

We feel that our data “suggest that a severe hearing loss may be associated with a reduced volume in the inferior colliculus in **aged humans**” (e.g. see Fig. 4 that shows that in the group with an age over 60 years the proportion of individuals with a high degree of hearing loss on one or both sides is significantly higher and IC volume on average reduced as compared to the group of younger adults).

As suggested by data presented in Fig. 5 that analyzed IC-volume from individuals with a pronounced asymmetrical hearing loss, the Wilcoxon test for the 13 available data points revealed a significantly smaller IC volume for the deprived as compared to the “normal” IC. However, this asymmetry was not apparent in the youngest individuals suggesting that deprivation does not lead to a shrinkage of IC volume in babies but rather may affect the normal age dependent growth. Consequently, we are carefully stating at the end of the introduction, that we cannot generally confirm that the IC volume correlates with hearing loss. Additional data from carefully selected sets of patients with defined age groups and hearing status would be necessary to evaluate the effect of hearing status on IC volume in more detail.

Motivated by your concerns, we have looked again at the 48 adult cases (>10yrs) and examined the correlation between IC volume and average PTA threshold of the contra-lateral ear. Because we obtained 2 related measurements (left and right IC) from 48 independent subjects in the present sample, the analysis was performed separately for the left and the right IC. The corresponding plots are shown in the diagrams below.

The linear correlation analysis of hearing loss in the contralateral ear as a function of IC-volume for the 48 adults over 10 years of age resulted for both ears in a regression line (left IC: y = -0.711x + 62.013; R² = 0.0246; right IC: y = -1.0015x + 69.305; R² = 0.0841) with a negative slope, suggesting that a small IC-volume tends to be associated with a high hearing loss and vice versa. In the present sample of adults over 10 year of age the effect of hearing loss was significant (p<0.05) for the right, but not for the left IC. The coefficients of determination show that the degree of hearing loss explains roughly 2.5% and 8.4% of IC-volume variation of the left and right IC respectively in this sample.

**I feel that the devinitiveness of the study findings should be presented consistently throughout the article, and I would recommend toning-down the speculative claims in the abstract as they are not representative of the article as a whole.**

We have presented our data, performed the statistical analysis, and are presenting conclusions based on statistically significant findings.

**It would be useful in the introduction to briefly define age-related hearing loss.**

We have added the following sentence in our introduction, line 46-47:

"Age-related hearing loss refers to a degenerative process of aging resulting in a progressive bilateral hearing loss. (Nelson EG, Hinojosa R. Laryngoscope 2006;116 (Suppl 112):1-12.)"

**What sort of thresholds are considered significant? Who’s definition is this? You can then refer to this in the methods section when defining loss in the context of the study.**

Our classification is in close accordance with the classification of the guidelines of the World Health Organization (2019). It combines the grades 0 (no impairment, <25dB) and 1 (slight impairment, 26-40dB) as category 1 (normal to mild hearing loss) and grade 4 (profound impairment including deafness, >81dB) as category 3 (severe hearing loss or deafness) as listed on page 14/15, lines 141-146.

**Why were undeveloped children (3 months up) included in the study? Would you not expect that the IC would still be increasing in size at that point?**

This is an explorative analysis of retrospective data to evaluate the feasibility of IC-volume measurements in routine clinical MRIs, and consequently we included the available MRIs independent of age and subsequently analyzed the effect of age.

**Do you have any evidence for a maximum IC size or an age at which the brain has reached its adult size in the majority of people?**

Fig. 2-4 show IC volume as a function of age. Due to the inter-individual IC-volume variation it is difficult to define an unequivocal maximum.

**I later see you used 10 years as the cut-off between childhood and adult – is there any evidence for the IC not increasing in size beyond 10 years old?**

The age distribution in Figs. 2-4 shows a clear age dependence of IC volume. There are no clear and obvious steps in this distribution of age and IC volume, however, the data show an initial increase with age, and overall a smaller volume in the oldest individuals. Due to the inter-individual variability of IC-volume age-groups were arbitrarily selected, based on the logarithmic age distribution in Figs. 2-4. The “naked eye” on the present set of data suggested no obvious growth in the group of babies before 1 year of age, an obvious increase between 1 and 10 years, a maximum between 10 and 60 years (mean age in the group of individuals between 10 and 60 years was ≈38 years) and a reduction above 60 years. This definition of age groups was not based on obvious or distinct discontinuities of IC volume with increasing age, rather it was used to describe general trends. In addition, the 10 year cut off is supported by the data on the development of the human auditory system presented by Moore and Linthicum (Int J Audiol, 2007.46(9):460-78. The human auditory system: A timeline of development.; “later childhood, from six to twelve years, is the time of maturation of the superficial cortical layers and their intracortical connections”).

**How was the edge of the IC determined? Please describe all methods used – was there a tissue contrast visible? Were some assumptions made about the internal anatomy of the brainstem?**

The interpretation of radiologic images in general requires a basic knowledge of anatomy and some experience. We feel that this is rather trivial and goes beyond the scope of the description in the methods.

As indicated in Fig. 1, the IC was located in orthogonal views of the brainstem using relevant land-marks like the cerebellum. The surface of the IC and the midbrain border of the IC could be distinguished after some training as illustrated in Fig. 1. Consequently, voxels belonging to the IC could be identified and marked as “region of interest” for volume determinations.

**The first mention of BERA (line 111) is not defined – but it is later.**

This has been corrected in line 113-114.

**Why was 6 months between audiometry and MRI used as the cutoff? Do you have any evidence supporting this or supporting not allowing a longer period of time between assessments? Broadening the period may introduce more noise into the associations but would it give you many more datapoints?**

The 6 months limit between audiometry and MRI was rather arbitrarily selected. This criterion did not exclude “many more data points” from the analysis. We excluded 9 cases where the time between MRI and audiogram was on average 2.6 years, which we believe weakens the correlation between IC-volume and threshold determined years later.

**Could you do a power calculation to see how many you would expect to need? If this could be done on a subset of the data, you may even be able to complete your new analysis with the remainder of the dataset.**

The present explorative and retrospective analysis provides the data that can be used to perform adequate power calculations to determine the required sample size for more detailed future studies.

As indicated above in response to the relation of hearing loss and IC-volume, the correlation of hearing loss and IC-volume revealed that hearing loss explains less than 10% of IC-volume variation. A power calculation indicates that a sample of approximately 150 subjects would be required to reach significance for a coefficient of determination of 0.05. Instead of using such a big sample with a highly variable degree and etiology of hearing loss, we suggest to examine the effect of hearing loss on IC-volume by comparing age matched groups with normal hearing and defined degrees of uni- and bi-lateral hearing loss.

**Please pull the MRI scanning parameters together more helpfully. You state that all images had the same matrix size (256*256*160) but later imply that the voxel sizes varied in all three dimensions. Is this the case? Were all scans definitely MPRAGEs or did the contrast and/or readout vary? Did all scans have square in-plane voxels? Why did the voxel size vary (I understand that this data is all from a single site – correct me if I am wrong)?**

In the supplementary material we now provide the relevant attributes from the DICOM header that result in variable voxel-volumes despite the matrix size of 256*256*160.

Two parameters affect the voxel volume for this matrix: slice thickness (DICOM attribute 0018,0050) and pixel spacing DICOM attribute 0028,0030). In small children up to 3 years slice thickness and pixel spacing were smaller than in adults, resulting in smaller voxel volumes. All the scans were MPRAGEs and all scans had square in-plane voxels.

**Was the protocol updated at a particular timepoint? Or do different protocols use different parameters – is it possible that the chosen protocol may be associated with the investigation of any other pathology (e.g., stroke, trauma, tumour)?**

We do not have this information and it is possible that the protocols may be associated with the investigation of other pathologies. The protocol recorded in the DICOM header did not change at a particular time point during the time when these data were collected, the main variation was with respect to slice thickness and pixel spacing.

**Were you able to see the reason that the MRI was requested? Was that associated in any way?**

Apart from young children and adults who received their MRI in the course of cochlear implantation diagnostics we were not able to identify the reason for MRI requests. There was no association regarding this issue.

**I had to re-read lines 130-132 a few times to understand what you meant. Please reword to clearly state that PTA was used in 67 cases and BERA in the remaining 56.**

We have expressed this sentence more clearly in lines 133-137.

**Why pool all 10-60 year olds into one large group? Surely it would be more useful to divide this group down? Please justify your methodology. Especially in light of your statement (lines 312-13) that maturation proceeds into the teenage years.**

Subdividing this group would result in 2 or more groups with a smaller sample size. The variation of volume with age appears obviously rather small in individuals varying in age between 10 and 60 years. Consequently, we do not really expect to demonstrate big differences in mean IC volume when comparing age subdivisions of the 10-60 year group, and the interpretation of (expected) non-significant differences between age-subdivisions will probably not result in important new insights.

**You mostly refer to gender throughout but male and female are sexes (man and woman are genders). It’s fine to say sex as this is medical records you’re dealing with. It’s more important to be consistent.**

We have changed the term to " gender "consistently in the whole manuscript.

**I disagree with the statement (lines 233-237) that the the proportion of individuals with a high degree of hearing loss on one or both sides being significantly lower in adults (10 to 60 years, 7 of 33) as compared to the old (>60 years, 8 of 15) individuals suggests that severe hearing loss (at least on one side) may be associated with a reduced IC-volume in aged humans.**

We show that the proportion of subjects with severe hearing loss is significantly higher (Fisher exact test) in the old as compared to the young adults (see line 239-243). We also show that IC-volume is significantly lower in the group of old as compared to the group of young adults (see line 179-180). These two significant findings, in the context of many published studies of the effect of deprivation on brain structure (our own animal studies: Gleich and Strutz, 1997 [cochlear nucleus]; Gleich et al., 2014 [inferior colliculus]; recent reviews from human studies, e.g.: Golub, 2017; Alfandari et al., 2018, Uchida et al., 2021), form the basis of our suggestion that severe hearing loss may be associated with the reduced IC volume in aged humans.

**If that were the case you would be able to test directly for the association between PTA/BERA and IC volume.**

See our response above where we now have plotted the correlations of hearing loss as a function of IC-volume for the 48 adults and provide an estimate for the required sample size for a coefficient of determination of 0.05. As described in lines 228-235 the heterogenous sample of this retrospective analysis with respect to hearing loss hampers the direct analysis of hearing loss vs. IC-volume and we suggest to examine the effect of hearing loss on IC-volume by comparing age matched groups with normal hearing and defined degrees of uni- and bi-lateral hearing loss to address this question in future studies.

**Your within-subject asymmetric hearing vs asymmetric IC volume finding is much more interesting and compelling.**

**I don’t fully understand why you have performed the comparisons you have, used the group definitions you have, and omitted what seems to be the most obvious tests.**

See the reasoning described above.

**Did you perform more tests with non-significant outcomes?**

We did not.

**All tests need to reported and if necessary multiple comparisons need to be corrected for.**

We have not performed multiple comparisons

**6. PLOS authors have the option to publish the peer review history of their article (what does this mean?). If published, this will include your full peer review and any attached files.**

We have no objections to publish the peer review history of our article.
