# Supplementary material for: Measurement of inferior colliculus volume based on MRI image stacks and its relationship with age and hearing status
Source: PLoS One. 2025 Jan 28;20(1):e0317363. doi: 10.1371/journal.pone.0317363 (PMC11774353; doi:10.1371/journal.pone.0317363)
Supplement: Attachment — Submitted filename: Response to Reviewers.docx [file pone.0317363.s003.docx]

Detailed response to the reviewers´ comments:

**Reviewer #1**

***Initially, I would like to express my displeasure at receiving a review without indications of modifications in the body of the text.***

As required for the revision, we submitted a marked-up copy of the manuscript named “revManuscript with tracking.docx” that highlights all the changes made to the original manuscript version. The modifications were indicated by the “markup function” of Microsoft Word. We have no explanation why the reviewer could not see the marked modifications made during the revision.

***Another point, the indication of the use of the classification of the degree of hearing loss is still not mentioned in the text. It's no use mentioning that you used the reference and not presenting this data to the reader.***

We now outline the definition of the different grades of hearing loss that were defined in the “Report of the Informal Working Group on Prevention of Deafness and Hearing Impairment Programme Planning, Geneva, 18-21 June 1991” in the methods and include this WHO report it in the references as number [29] “World Health Organization. (1991). Report of the informal working group on prevention of deafness and hearing impairment programme planning: Geneva, 18–21 June 1991. Geneva: World Health Organization. Retrieved from https://apps.who.int/iris/handle/10665/58839.”

Our classification is based on the grades defined in the report of the World Health Organization. (1991). To reduce the number of groups and increase the number of samples within each group for statistical analysis, we defined 3 categories by combining the grades 0 (no impairment, <25dB) and 1 (slight impairment, 26-40dB) as category 1 (≤40dB), grades 2 (moderate impairment, 41-60dB) and 3 (severe impairment, 61-80dB) as category 2 (41-80dB) and grade 4 (profound impairment including deafness, >81dB) as category 3 (see now end of method section).

***Regarding the use of the 5kHz stimulus, it is well known that the Biologic equipment performs the analysis at this frequency in all its versions. And it is important to say that there is no recommendation for its use at 5kHz and there never has been. Concurrently, this frequency is not analyzed in the PTA. Therefore, the responses are not suitable for publication.***

As explained in the initial revision, this study is based on a retrospective analysis of cases from the ENT department of the University of Regensburg. BERA is routinely used in our clinic to diagnose hearing status at 3 frequencies in babies where PTA is not possible. Before 2017 the high frequency tested was 5 kHz which was changed to 4 kHz after 2017.

We disagree with the reviewer´s notion that the determination of the hearing status by BERA can only be evaluated with a test frequency of 4 kHz but not with 5 kHz. Assuming a 35mm long basilar membrane, the position of the 4 kHz place from the cochlear base is ≈11.6 mm or 33% while that of the 5 kHz place is ≈10.0 mm or 29% from the base (according to Greenwood, 1961). Thus, based on the logarithmic frequency representation along the human cochlea, the difference between these two frequencies is not dramatic. In addition the hearing class is based on the average threshold of the 3 BERA frequencies tested. We do not see that the BERA determination of threshold at 5 kHz versus 4 kHz would substantially affect the outcome of the hearing loss classification.

***The responses are vague and the insertions made did not significantly improve the quality of the manuscript. Therefore, I do not recommend the publication of the manuscript.***

In the first revision we comprehensively answered and discussed 16 points raised by the reviewer and made corresponding changes and additions to the manuscript.

The points initially raised by the reviewer and our responses are repeated below. We regard these responses as specific and detailed and believe that the corresponding changes to the manuscript improved the manuscript. We disagree with the global classification of our responses to the 16 points raised in the first review by this reviewer as “vague”.

**“You mentioned that all patients have PTA or BERA. However, it is crucial to specify the type of BERA that was employed, as the BERA-click does not provide data that is comparable to PTA. Therefore, examine this item and substitute the procedure name for BERA-specific frequency.**

For the present exploratory retrospective analysis of the relation of hearing status and IC volume we noticed early during the study the heterogeneity of the subjects with regard to age and hearing status (e.g. Fig. 4). Thus, we focused on a coarse classification of hearing loss that can be achieved by evaluating standard clinical PTAs and BERAS performed in the audiology section of the Regensburg ENT clinic.

To obtain frequency specific information we recorded in Regensburg the response to short tone bursts which were used to evaluate the hearing status. For measuring the interpeak-latences I-V click stimuli were used.

We have added a sentence concerning this issue in lines 137-139 of the manuscript.

**PTA – indicating the values and equipment that are used.**

See Raw data (S1 Table) that are now provided in “supplementary data” and give the thresholds obtained at the PTA test-frequencies and for the BERA stimuli.

The equipment used for PTA: Madsen Astera, Co. Otometrics/Münster-Germany

The equipment used for BERA: Navigator Pro, Bio-Logic/Natus, Model 580-NVBOX1156A, Worthing, UK.

**The BERA frequency specifies the values and equipment that are employed. Before the specified frequency was reached, BERA was executed–click?**

To obtain frequency specific information we recorded in Regensburg the response to short tone bursts which were used to evaluate the hearing status. For measuring the interpeak-latences I-V click stimuli were used.

**Why was 5kHz employed? This frequency is not of great significance within an audio battery.**

For the BERA values in this study, the Navigator Pro-machine was used which had been preset for the examination with click-stimuli using the upper frequency limit at 5kHz.

In our newer BERA equipment (Eclipse by Interacoustics, Middelfart, Denmark) the upper frequency limit for clicks is now 4kHz.

However, as this is a retrospective study, the examination frequency cannot be changed to 4kHz. Consequently, high frequency loss in the BERA measurements was determined in the majority of earlier data by 5kHz and in more recent measurements with a 4kHz setting.

**It is strongly recommended that auditory loss be classified in accordance with the guidelines of the World Health Organization (2019). And that the categories be divided by this classification.**

Our classification is in close accordance with the classification mentioned above. It combines the grades 0 (no impairment, <25dB) and 1 (slight impairment, 26-40dB) as category 1 (normal to mild hearing loss) and grade 4 (profound impairment including deafness, >81dB) as category 3 (severe hearing loss or deafness) as listed in lines 148-154.

**I would have appreciated the opportunity to review a prior study that examined the number of participants who would be considered reliable for this study.**

Since this exploratory retrospective study was, to our knowledge, the first systematic trying to correlate the relation of IC-volume with other factors like age or hearing status, these data can provide the basis to estimate the required sample size for more detailed future studies.

**Note that the size of the letters is greater than the remainder of the manuscript on page 10, lines 182 to 184.**

This formatting mistake has been corrected. Due to corrections in the manuscript the lines have shifted to 189-191.

**It is essential to mention in a specific section of the manuscript that the PTA is the standard for diagnosing auditory loss in children who are capable of responding appropriately to the elderly.**

PTA was used in older children and adults when possible. BERA was used in babies when a PTA could not be reliably measured. In some deaf children over 1 year a BERA was performed in the course of Cochlear Implant diagnostics.

This we now mentioned in the methods part lines 132-137:

The PTAs are the standard for diagnosing auditory loss in children who are capable of responding appropriately to the examiner. PTA was used in 67 children and adults and determined air-conduction thresholds using 9 frequencies (125Hz to 8kHz). The BERA was used in 56 young children (aged 3 months to 4 years) in whom a PTA could not be reliably measured, and at least 3 frequencies were included (500-1000Hz, 2kHz, 5kHz). In some deaf children over 1 year of age, a BERA had been performed in the course of cochlear implant diagnostics.

**In the discussion, the first sentence indicates that hearing loss is more prevalent after the age of 70. I suggest reading additional articles, as there is already evidence that it occurs more frequently today.**

The National Institute on Deafness and Other Communication Disorders states in their Quick Statistics about Hearing, Balance & Dizziness Report which was updated Sept. 20, 2024, that among adults ages 20-69 who report 5 or more years of exposure to very loud noise at work, about 18% have speech-frequency hearing loss in both ears. About 5% of adults ages 45-54 have disabling hearing loss. The rate increases to 10% for adults ages 55-64. 22% of those ages 65-74 and 55% of those who are 75 and older have disabling hearing loss.

These numbers do not differ so much from the ones stated in our discussion.

Another study from 2010 looked at hearing thresholds from 1999-2004 and compared them with thresholds from 1959-1962 ( Hoffman HJ et al. Americans hear as well or better today compared with 40 years ago: hearing threshold levels in the unscreened adult population of the United States, 1959-1962 and 1999-2004. Ear Hear 2010 Dec;31(6):725-34.) They found that prevalence of overall hearing impairment was significantly lower in 1999-2004 than in 1959-1962.

However, the population worldwide is becoming older and, therefore, age-related hearing loss can be found more frequently than in the past.

**Additionally, I suggest that the discussion regarding prevalence and incidence be more in-depth, including, for instance, the fact that adolescents and children tend to lose their hearing even more rapidly as a result of the use of hearing aids. The initial paragraph is extremely rudimentary and inadequate.**

The aim of this study was to examine the volumes of the inferior colliculus and relate them with age and hearing loss. A discussion regarding prevalence of hearing loss and the impact of the use of hearing aids on hearing in adolescents would be suitable for a review study but not for this retrospective analysis, which cannot find answers for the changes in prevalence of hearing loss.

**In terms of the IC volume, it was determined that two individuals exhibited values that were higher. I suggest that they draw conclusions about this. What was the profession of these individuals? Were they musicians? In conclusion, they must better package this item.**

This is an interesting point and we looked up these two cases.

Both patients with the high IC volumes happened to be deaf children (3 months and 4 years old), so they were certainly not musicians. We could not draw any sound conclusions from the results.

**Furthermore, when discussing the IC volume in relation to left/right, they also mentioned left/right-handedness very appropriately. However, did you analyze this variable?**

Unfortunately, we have no records of the handedness.

**It would be an extremely significant point in this study. Given the impossibility of this analysis, as it is a retrospective study, I suggest that it be included as a constraint on the study.**

The following sentence was added to the discussion lines 387-388

It is a constraint of this study, that handedness cannot not be considered in the discussion of the IC volume in relation to right/left sides (lateralization of hearing), as there were no records available.

**I suggest that the study's limiting factors, which encompass a multitude of other factors, be updated, and that new studies be proposed.**

This sentence was added at the end of the discussion line 416-418:

Since this exploratory retrospective study was, to our knowledge, the first systematic trying to correlate the relation of IC-volume with other factors like age or hearing status, these data can provide the basis for focused study designs comparing age matched groups with well-defined hearing status and for the estimation of the required sample size for more detailed future studies.

**Furthermore, it is not possible to generalize and assert that there is no symmetry, which would imply that this was observed in the current study with a reduced number of participants.**

A Wilcoxon paired sample test comparing left and right IC volume of the 123 subjects found no systematic significant difference or systematic asymmetry between both sides (p = 0.865).

We do not state in the manuscript that “there is no symmetry”, rather we describe that the data “show no systematic difference … between both sides.” and this is also reflected in the discussion lines 381 – 386.

**The same applies to the other apprehensions. This study must be replicated with an appropriate number of participants, as well as more clearly defined evaluation procedures.**

We agree that our retrospective explorative analysis cannot unequivocally answer many detailed questions. But it provides the basis for the focused design of future studies with adequate estimates of required sample size.”

As illustrated by the answers above, we do not consider our responses to the questions raised by the reviewer in the first revision as “vague”.

**Reviewer #2:**

***I have one further comment and suggestion to make on the subject of describing in your methods section how the edge of the IC was determined. I believe it is the responsibility of authors to describe all methods used such that they can be replicated by a reader. I do not believe this is "trivial" or that it "goes beyond the scope of the description in the methods".***

***Please *briefly* describe the process used for drawing the ROI so that a reader could try and follow the process, or cite an article that does so.***

The corpora quadrigemina consist of the inferior (more caudal) and superior (more rostral) colliculi and are elevations of the tectum forming the dorsal surface of the midbrain. As illustrated in the original orthogonal MRI views in Fig. 1A, the surface of the IC is well defined and clearly recognizable. The body of the IC below its surface appears as brighter area of the tectum. Fig. 1B illustrates how voxels within these borders of the IC were manually labelled as "Region of Interest" to determine the number of voxels belonging to the left and right IC (Fig.1A and Fig.1B).

This description has been added to the method section.
